# A retrospective study of 51 pediatric cases of traumatic asphyxia

**DOI:** 10.1186/s13019-022-01773-2

**Published:** 2022-03-13

**Authors:** Hui-rong Luo, Xuan Zhai, Si-min Xie, Xin Jin

**Affiliations:** 1 Department of Psychiatry, The First Affiliated Hospital of Chongqing Medical University, Chongqing, People’s Republic of China; 2 Department of Surgical Emergency Center, Children’s Hospital of Chongqing Medical University, Chongqing, People’s Republic of China; 3 Department of Orthopedic Surgery, The First Affiliated Hospital of Chongqing Medical University, Chongqing, People’s Republic of China; 4 Department of Surgical Emergency Center, Ministry of Education Key Laboratory of Child Development and Disorders, National Clinical Research Center for Child Health and Disorders, China International Science and Technology Cooperation Base of Child Development and Critical Disorders, Chongqing Key Laboratory of Pediatrics, Children’s Hospital of Chongqing Medical University, Chongqing, People’s Republic of China

**Keywords:** Traumatic asphyxia, Pediatric, Thoracic trauma

## Abstract

**Background:**

Traumatic asphyxia (TA) is a rarely reported disease characterized as thoraco-cervico-facial petechiae, facial edema and cyanosis, subconjunctival hemorrhage and neurological symptoms. This study aimed to report 51 children of TA at the pediatric medical center of west China.

**Methods:**

Scanned medical reports were reviewed and specific variables as age, sex, cause of injury, clinical manifestations and associated injuries were analyzed using SPSS 25.0.

**Results:**

The average age of patients was 5.3 ± 2.9 (1.3–13.2) year-old. Thirty (58.8%) were boys and 21 (41.2%) were girls. Most TAs occurred during vehicle accident, object compression and stampede. All patients showed facial petechiae (100.0%, CI 93.0–100.0%), 25 (49.0%, CI 34.8–63.2%) out of 51 presented with facial edema, 29 (56.9%, CI 42.8–70.9%) presented with subconjunctival hemorrhage, including bilateral 27 and unilateral 2. Six patients had facial cyanosis (11.8%, CI 2.6–20.9%). Other symptoms were also presented as epileptic seizure, vomiting, incontinence, paraplegia, etc. The most frequent companion injury was pulmonary contusion (76.5%, CI 64.4–88.5%). Other companion injuries included mediastinal emphysema, fracture, cerebral contusion and hemorrhage, hypoxic-ischemic brain injury, abdominal organ contusion, mastoid hemorrhage, hematocele of paranasal sinuses, spinal cord injury, hepatic insufficiency, myocardial injury and retinal hemorrhage and edema. Treatment was mainly supportive. No death occurred in our study. The prognosis is rather good if without damage of central nervous system.

**Conclusion:**

TA could bring out multiple symptoms, among which retinal hemorrhage and edema, spinal cord injury and viscera impairment have been less observed. Comprehensive physical and auxiliary examination should be performed considering TA. Its prognosis is rather good with focus on life-threatening complications.

## Introduction

Firstly described in 1837, traumatic asphyxia is characterized as thoraco-cervico-facial petechiae, facial edema and cyanosis, subconjunctival hemorrhage and neurological symptoms [1]. TA could be triggered with or without external forces, so it also bears the name as compression asphyxia and crush asphyxia [2]. The incidence of TA was unclear with possible underestimation [3]. Usually caused by vehicle rolling over, object compression, it could also be presented from entrapment and uncontrolled crowd as Hillsborough tragedy [4]. The treatment is supportive with focus on severe associated injuries. Here we reported 51 pediatric TA cases presented for treatment during May 2005 and November 2020 at the pediatric medical center of west China.

## Methods

This retrospective study evaluated records of 51 children of TA between May 2005 and November 2020 at the pediatric medical center of west China. Inclusion criteria: (1) TA typically occurs as petechiae in the face, neck and chest or conjunctival hemorrhage or facial edema and cyanosis; (2) Diagnosed as “traumatic asphyxia”, “thoracic crush syndrome” or “superior vena cava crush syndrome” during hospitalization; (3) Admission within 10 days after trauma; Exclusion Criteria: (1) Incomplete medical records; (2) inconsistency between symptoms and examination results; (3) Outpatient medical records; (4) Lost to follow-up.

Data collection followed previously published guidelines on retrospective chart review [5]. The electronic medical record browser of patients’ hospitalization and the platform of big data in our hospital were used to search for all TA patients. Without blindness, two already trained abstractors (HRL, XJ) abstracted data from the scanned medical records, including physician and nursing notes and auxiliary examinations.

This study recorded the following variables: age, sex, cause of injury, department of admission, way of admission, vital signs in admission, past medical history, initial Glasgow Coma Score (GCS), loss of consciousness, initial CT results of head, chest, abdomen and extremities, MRI results of brain and spinal cord, testing results of myocardial markers and liver function, routine urine test. Symptoms as facial edema and cyanosis, petechiae on the face, neck and chest, subconjunctival hemorrhage, hemorrhage of ear, nose and oral mucosa, vomiting, seizure, incontinence, retinal hemorrhage and edema were also recorded. Hospital length of stay (LOS) and pediatric intensive care unit (PICU) stay were also recorded. Other records were viewed without a standardized form, such as companion injuries, treatment with drugs, surgeries, blood infusion and other interventions.

SPSS 25.0 (IBM SPSS Statistics for Windows, Version 25.0, Armonk, NY) was applied for data analysis to calculate 95% confidence interval (CI). Normal approximation intervals (standard CIs) were calculated for proportions if n ≥ 30 and np > 5 and n (1 − p) > 5. Exact 95% CIs (Clopper-Pearson Confidence Intervals) were used for smaller samples. Mean ± standard deviation (Mean ± SD) was used to describe age. Range (Min–Max, Median) was used for LOS and PICU stay. Categorical data were analyzed with Fisher’s exact test in cases of small cell size. A Krustal-Wallis test was used to compare quantitative data which were not normally distributed. *P* < 0.05 was deemed statistically significant.

This retrospective study has been conducted with approval of the Ethics Committee of the Children's Hospital of Chongqing Medical University (Approval Number: (2021) Institutional Review Board (IRB) (STUDY) No.44).

## Results

### Characteristics

Through rough research, we collected data of 87 patients with TA diagnosis, including 20 children from outpatient, 8 repeated data of patient, 3 patients of inconsistent symptoms with TA diagnosis, 2 patients with incomplete hospitalized data, 2 patients admitted in 10 days after trauma and 1 patient discharged automatically without proper treatment. Finally, 51 children were included in our study. They were admitted with TA between May 2005 and November 2020 at the pediatric medical center of west China. Thirty (58.8%) were boys and 21 (41.2%) were girls. Forty-one cases were transferred in from inferior hospitals, 5 were admitted from outpatient department and 5 were from emergency department. Their average age was 5.3 ± 2.9 (1–13) years old. The causes of TA were traffic accident in 34 cases, object compression in 10, stampede in 5, knife stab in 1 and falling from high and compressed by an adult in 1. The descriptive features of the causes were presented in Table 1. Grouped by cause of injury, only ages were statistically different. 35 out of 51 (68.63%) had vital signs that were within normal limits. During admission, apart from a patient by knife stab with vital signs as unattainable temperature and blood pressure, pulse of 25 bpm, respiration of 20 under respiratory machinery, and oxygen saturation as 25%, 4 children got fever over 37.3 °C, 11 children got respiration more than 30 times per minute with highest as 45 times per minute, and 5 children got heart rate over 130 bpm with the highest as 170 bpm. All patients got a clean past medical history.

**Table 1:** Causes of injury, age, gender, and length of stay (LOS). Categorized by causes of injury, only age is statistically significant, which is probably due to that all children with stampede in school was injured during the same event after attending an final examination

Causes of injury	Total	Age (year)	Sex		LOS (day)
	n (%)	Mean ± SD	Male	Female	Min–Max (Median)
			n (%)	n (%)	
Total	51 (100.0%)	5.3 ± 2.9	30 (58.8%)	21 (41.2%)	3–73 (15)
*Traffic accident*	34 (66.7%)	4.9 ± 2.3	20 (66.7%)	14 (66.7%)	5–47 (15)
Vehicle roll-over	30 (88.2%)	4.9 ± 2.4	19 (95.0%)	12 (85.7%)	5–42 (15)
Inside the vehicle	3 (8.8%)	4.6 ± 1.5	1 (5.0%)	2 (14.3%)	14–47 (20)
Compressed between vehicles	1 (2.9%)	6.6 ± 0.0	0 (0.0%)	1 (7.1%)	13
*Object compression*	10 (19.6%)	4.0 ± 2.6	6 (20.0%)	4 (19.0%)	3–73 (10)
Object tip-over	3 (30.0%)	3.5 ± 3.3	2 (33.3%)	1 (25.0%)	6–12 (6)
Entrapment in the escalator	1 (10.0%)	3.2 ± 0.0	1 (16.7%)	0 (0.0%)	3
Entrapment in concrete mixer	1 (10.0%)	4.4 ± 0.0	1 (16.7%)	0 (0.0%)	55
Collapsed wall	1 (10.0%)	2.3 ± 0.0	1 (16.7%)	0 (0.0%)	19
Object or wound falling	2 (20.0%)	5.0 ± 0.0	1 (16.7%)	0 (0.0%)	11–73 (42)
Compressed by folding door	1 (10.0%)	3.3 ± 0.0	0 (0.0%)	1 (100.0%)	5
*Stampede*	5 (9.8%)	9.3 ± 0.6	3 (10.0%)	2 (9.5%)	10–30 (12)
Stampede in school	5 (100.0%)	9.3 ± 0.6	3 (100.0%)	2 (100.0%)	10–30 (12)
*Other causes of injury*	2 (3.9%)	7.9 ± 7.4	1 (3.3%)	1 (4.8%)	24–44 (34)
Knife stab	1 (50.0%)	13.2 ± 0.0	1 (100%)	0 (0.0%)	24
Falling from high and compressed by father	1 (50.0%)	2.7 ± 0.0	0 (0.0%)	1 (100%)	44
*P*		0.011^a^*	1.000^b^		0.309^a^

^*^p < 0.05

^a^Kruskal Wallis test

^b^Fisher Freeman Halton Test

### Symptoms

**Fig. 1:**
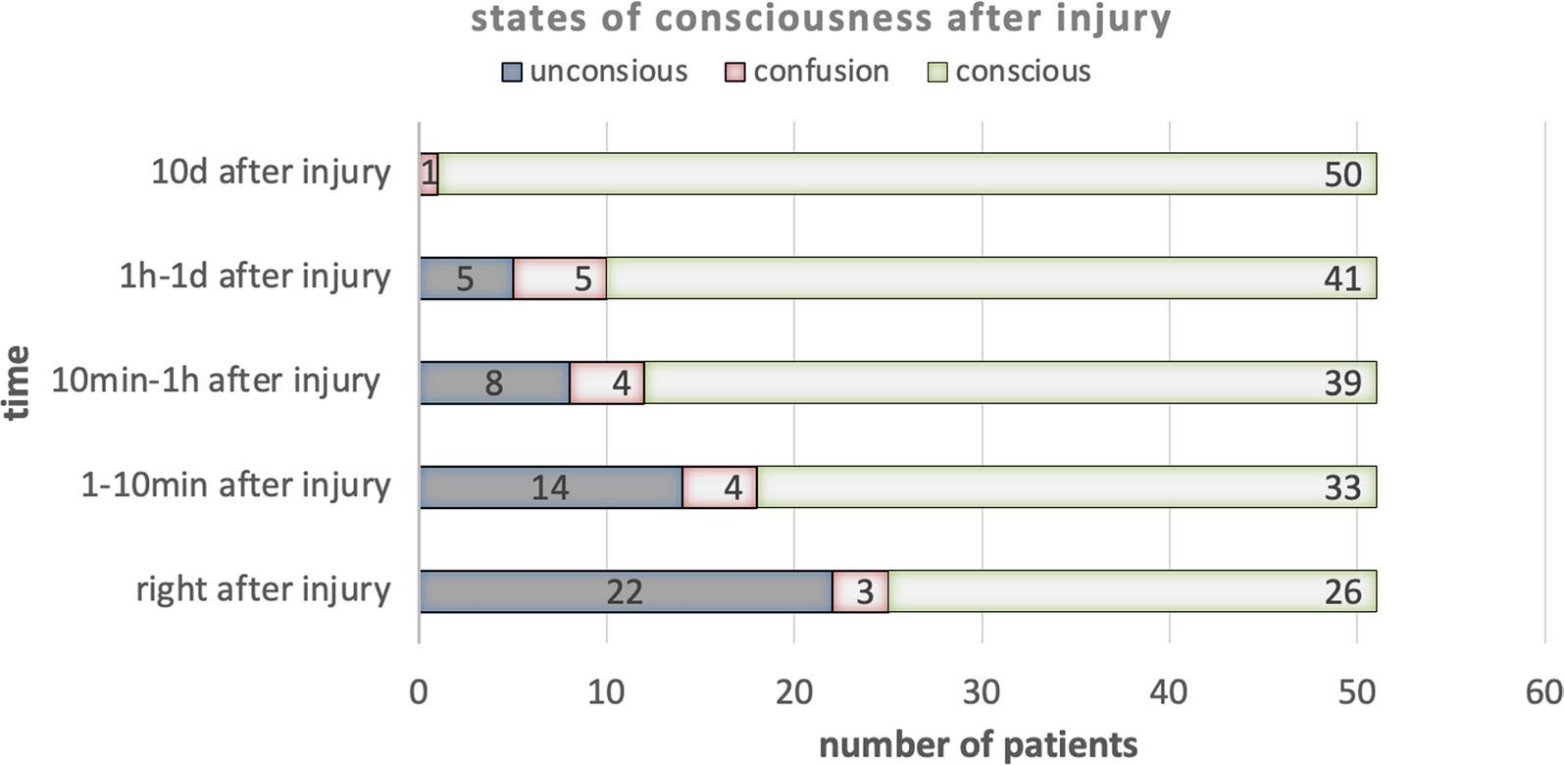
States of consciousness after injury. With time, unconscious patients decreased from 22 right after injury to 0 in 10 days after injury, consious patients increased from 26 right after injury to 50 in 10 days after injury. A transitional period of confusion was also observed

All patients (100.0%, CI 93.0–100.0%) showed petechiae on the upper body, especially on face. Twenty-five (49.0%, CI 34.8–63.2%) out of 51 presented with facial edema. As for subconjunctival hemorrhage, 29 (56.9%, CI 42.8–70.9%) presented with subconjunctival hemorrhage, including bilateral 27 and unilateral 2. Six patients had facial cyanosis (11.8%, CI 2.6–20.9%). Twenty-two children went through loss of consciousness within minutes or days, 3 children got confusion within days, and the other 26 remained conscious after injury. The change of neurological states with time was shown in Table 1. During admission, 82.4% (42/51) of patients were of GCS 15, 7.8% (4/51) were of GCS 13–14, 7.8% (4/51) were of GCS 4–7. Patients’ states of consciousness after injury were shown in Fig. 1. Six patients went through epileptic seizure, 5 vomited, 11 patients got incontinence after injury and 2 presented with paraplegia. Patients also showed nasal hemorrhage in 7, ear hemorrhage in 5, oral mucosa hemorrhage in 2 and hemoptysis in 1. Other symptoms included 2 cardiac arrest within minutes after injury, exophthalmos in 1 and blurred vision in 1. All symptoms were presented in Table 2 with numbers and percentages.

**Table 2:** Symptoms

Symptoms	N	Percentage (%)	Confidence interval (CI) (%)
*Common symptoms*	51	100.0	93.0–100.0
Facial petechiae	51	100.0	93.0–100.0
Subconjunctival hemorrhage	29	56.9	42.8–70.9
Facial edema	25	49.0	34.8–63.2
Facial cyanosis	6	11.8	2.6–20.9
*Neurological symptoms*	16	31.4	18.2–44.6
Epileptic seizure	6	11.8	2.6–20.9
Vomiting	5	9.8	1.4–18.3
Incontinence	11	21.6	9.9–33.3
Paraplegia	2	3.85	1.4–13.6
*Hemorrhage*	13	25.5	13.1–37.9
Nose	7	13.5	3.9–23.1
Ear	5	9.8	1.4–18.3
Oral mucosa	2	3.85	1.4–13.6
Hemoptysis	1	1.96	0.04–10.45
*Other symptoms*	3	5.88	1.23–16.24
Cardiac arrest	2	3.85	1.4–13.6
Blurred vision	1	1.96	0.04–10.45
Exophthalmos	1	1.96	0.04–10.45

### Companion injuries

All patients had CT scan of head, thorax and abdomen within 24 h of injury. Some also got results of MRI, ultrasonic inspection and eye fundus. Based on all those images and laboratory tests, only 3 patients got no companion injuries except skin abrasion. Companion injuries were presented as thoracic, craniocerebral, abdominal, craniocerebral, and other injuries as shown in Table 3.

**Table 3:** Companion injuries of TA

Companion injury	With	Without	Total	Percentage (%)	Confidence interval (CI) (%)
*Thoracic injury*	39	12	51	76.5	64.4–88.5^a^
Pulmonary contusion	39	12	51	76.5	64.4–88.5^a^
Mediastinal emphysema	5	46	51	9.8	1.4–18.3^b^
Multiple rib fracture	7	44	51	13.5	3.9–23.1^a^
Single rib fracture	2	49	51	3.85	1.4–13.6^b^
Clavicle fracture	6	45	51	11.8	2.6–20.9^a^
*Craniocerebral injury*	17	34	51	33.3	19.9–46.7^a^
Cranial fracture	8	43	51	15.7	5.4–26.0^a^
Cerebral contusion or hemorrhage	6	45	51	11.8	2.6–20.9 ^a^
Hypoxic-ischemic brain injury	8	43	51	15.7	5.4–26.0^a^
*Abdominal injury*	10	41	51	19.6	8.3–30.9^a^
Liver contusion	6	45	51	11.8	2.6–20.9^a^
Kidney contusion	5	46	51	9.8	1.4–18.3^b^
Spleen contusion	2	49	51	3.9	1.4–13.6^b^
Pancreas contusion	1	50	51	2.0	0.0–10.5^b^
Adrenal contusion	1	50	51	2.0	0.0–10.5^b^
*Other*	42	9	51	82.4	71.5–93.2^a^
Other fracture	22	29	51	43.1	29.1–57.2^a^
Mastoid hemorrhage	7	44	51	13.5	3.9–23.1^a^
Hematocele of paranasal sinuses	10	41	51	19.6	8.3–30.9^a^
Strainghtening or reversal of cervical lordosis	3	48	51	5.9	1.2–16.2^b^
Atlantoaxial subluxation	3	48	51	5.9	1.2–16.2^b^
Spinal injury	3	48	51	5.9	1.2–16.2^b^
Hematuria	6	45	51	11.8	2.6–20.9^a^
Hepatic insufficiency*	26	23	49	53.1	38.6–67.5^a^
Myocardial injury**	26	9	35	74.3	59.1–89.5^a^
Retinal hemorrhage and edema	3	12	15	20.0	4.3–48.1^b^

^*^Hepatic insufficiency defined as elevation of glutamic-pyruvic transaminase, glutamic oxaloacetic transaminase and lactate dehydrogenase within 2 days after hospitalization. Abnormal hepatic function caused by definitive liver contusion was not excluded

^**^Myocardial injury defined as elevation of creatine kinase isoenzyme-MB (CK-MB) and cardiac troponin (cTn) within 3 days after hospitalization

^a^Kruskal Wallis test

^b^Fisher Freeman Halton Test

Thirty-nine (76.5%, CI 64.4–88.5%) patients got pulmonary contusion (bilateral in 31 and unilateral in 8) with varying degrees of hemothorax and pneumothorax. Five patients (9.8%, CI 1.4–18.3%) got mediastinal emphysema. Seven patients (13.5%, CI 3.9–23.1%) got multiple rib fracture, 2 patients (3.9%, CI 1.4–13.6%) got single rib fracture. Six patients (11.8%, CI 2.6–20.9%) got clavicle fracture. Eight patients (15.7%, CI 5.4–26.0%) showed cranial fracture and 6 patients (11.8%, CI 2.6–20.9%) got cerebral contusion or hemorrhage, including 1 cerebral contusion and subarachnoid hemorrhage, 1 cerebral contusion and subdural hemorrhage, 2 cerebral contusion and 1 epidural hemorrhage. Ten patients (19.6%, CI 8.3–30.9%) got abdominal injury, including liver contusion in 6 (11.8% CI 2.6–20.9%), kidney contusion in 5 (9.8%, CI 1.4–18.3%), spleen contusion in 2 (3.9%, CI 1.4–13.6%), pancreas injury in 1 (2.0%, CI 0.0–10.5%) and adrenal injury in 1 (2.0%, CI 0.0–10.5%).

**Fig. 2:**
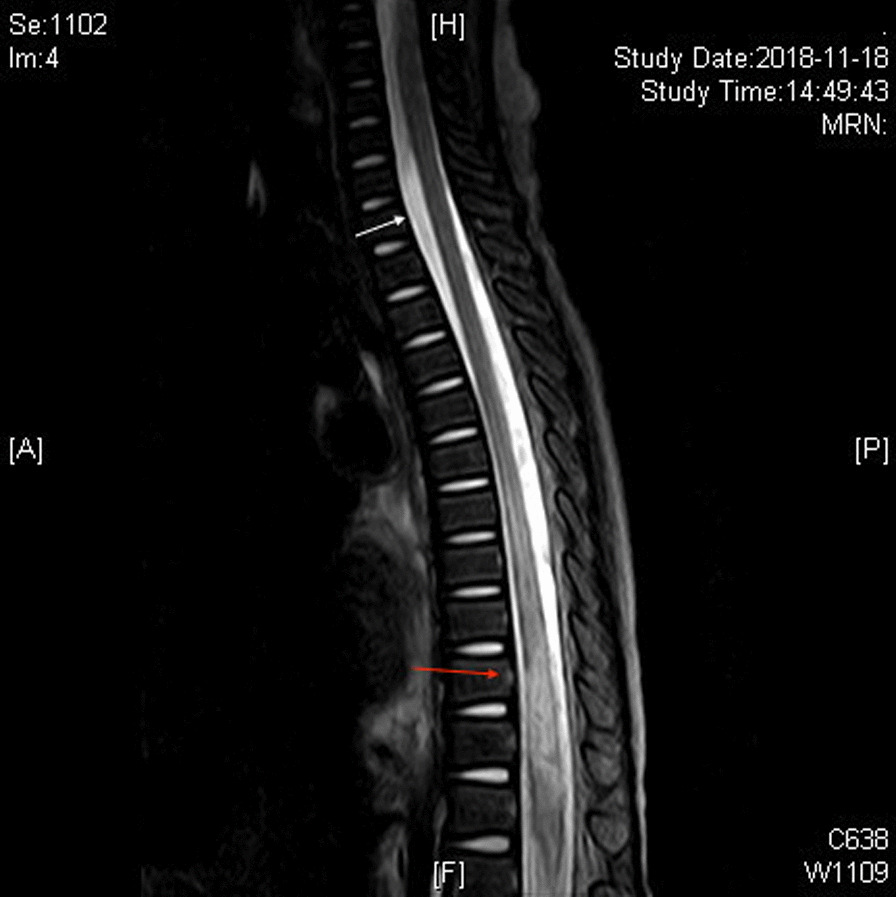
Sagittal T2-weighted magnetic resonance imaging (MRI T2W1) of injured spinal cord. There showed some patchy abnormal shadows with a hyperintense signal of spinal cord below thoracic 1 vertebrae (white arrow), indicating the possibility of spinal cord edema or contusion. There showed also spinal cord swelling at the level of thoracic 10–12 vertebrae (red arrow) with spine fracture, which indicated spinal cord injury (ASIA-A)

Twenty-two patients (43.1%, CI 29.1–57.2%) got other kinds of fractures including fracture in extremities, pelvis, scapula and vertebrae. Seven patients (13.5%, CI 3.9–23.1%) got mastoid hemorrhage and 10 (19.6%, CI 8.3–30.9%) got hematocele of paranasal sinuses. Three patients (5.9%, CI 1.2–16.2%) got strainghtening or reversal of cervical lordosis. Three patients (5.9%, CI 1.2–16.2%) got atlantoaxial subluxation. There showed 3 cases of spinal cord injuries with spinal MRI characterized as abnormal signal as edema and hemorrhage between or below specific thoracic fragments, among whom paraplegia was also noted as temporary or possibly permanent. One Sagittal T2-weighted magnetic resonance imaging (MRI T2W1) of injured spinal cord was presented in Fig. 2. There was also evidence of hematuria in 6 patients (11.8%, CI 2.6–20.9%). With insufficiency of specific examinations, we found 26 out of 35 (74.3%, CI 59.1–89.5%) presenting with myocardial injury defined as elevation of creatine kinase isoenzyme-MB (CK-MB) and cardiac troponin (cTn) within 3 days after hospitalization and 26 out of 49 (53.1%, CI 38.6–67.5%) showing hepatic insufficiency defined as elevation of glutamic-pyruvic transaminase, glutamic oxaloacetic transaminase and lactate dehydrogenase within 2 days after hospitalization without considering abnormal hepatic function caused by definitive liver contusion.

**Fig. 3:**
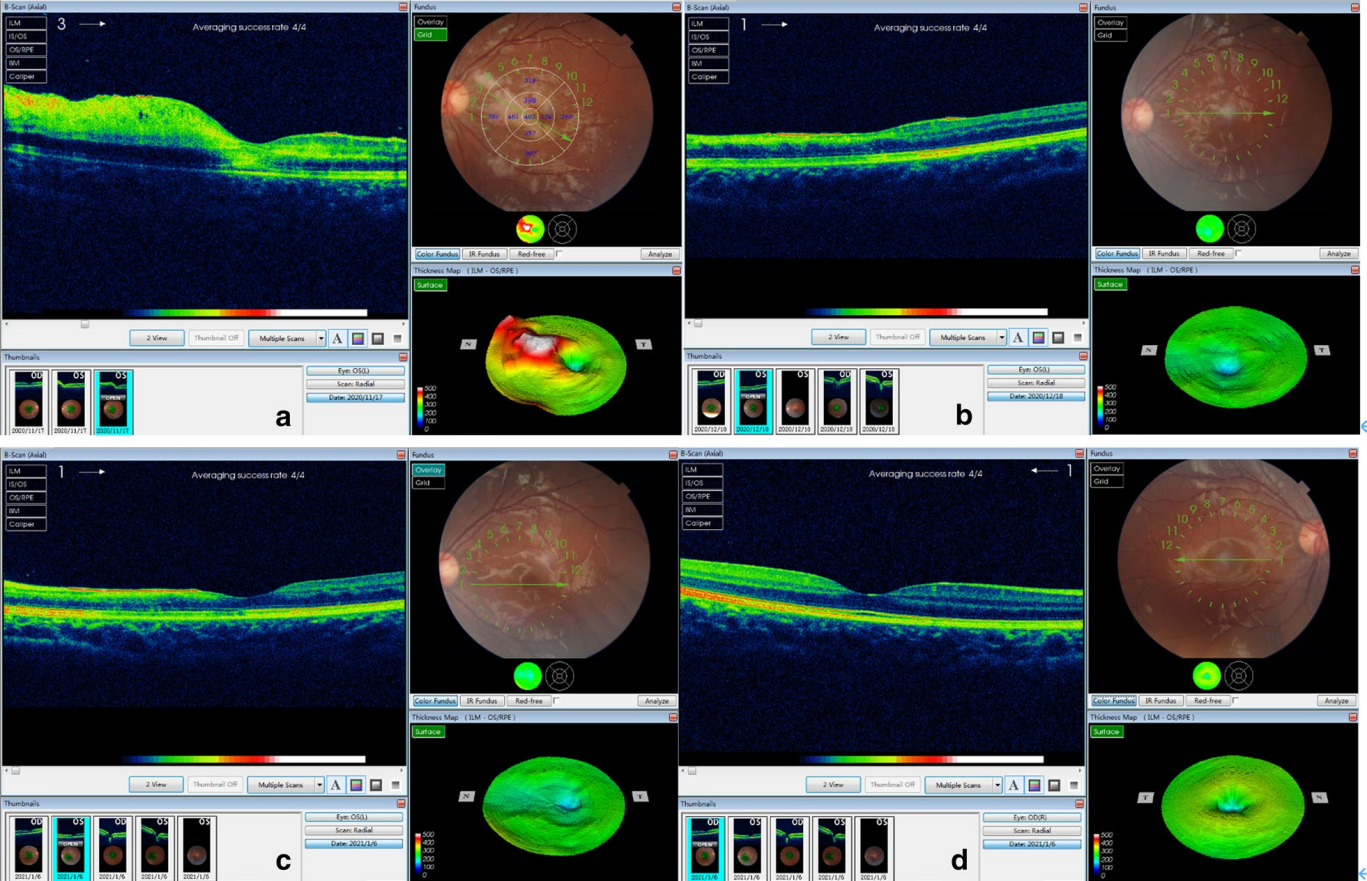
Optical coherent tomography (OCT), fundus and thickness map results of left eye of a TA patient during hospitalization (**a**), left eye in 1-month follow-up (**b**), left eye 1.5-month follow-up (**c**) and right eye in 1.5-month follow-up (**d**). Shown in **a**, the retina was hemorrhagic with edema, thickening and exudates around the optic disc in the left eye. The optic disc was pushed temporally with possible optic nerve compression. However, this child did not report about visual change at that time. In the 1-month and 1.5-month follow up as shown in **b** and **c**, the retina turned atrophy and the optic disc was drafted. These retinal changes are irreversible and responsible for lifelong amblyopia. **d** Presented the right eye in 1.5-month follow-up without obvious retinal changes

We also found 3 retinal hemorrhage and edema from 15 eye fundus (20.0%, CI 4.3–48.1%), among which the Optical Coherent Tomography (OCT), eye fundus and thickness map was presented in Fig. 3.

### Treatment and prognosis

Patients were hospitalized into different departments, including 19 in neurosurgery department, 15 in cardiothoracic surgery, 6 in orthopedics, 5 in hepatobiliary surgery, 4 in critical medicine, 1 in traumatology and 1 in burn and plastic surgery, among whom 3 patients were transferred into rehabilitation department for further treatment because of spinal cord injury in 2 and severe cerebral ischemia in 1. The LOS of all patients was 3–73 days (Median 15 days). Ten patients went into PICU for 1–43 days (Median 4 days). All patients got supportive treatment as hemostatic drugs, oxygen uptake and stay in bed with raised bed head by 15–30°. The 2 arrested patients received successful cardiopulmonary resuscitation. Two patients received noninvasive ventilator assisted ventilation, 7 patients received invasive ventilator assisted ventilation and positive end expiratory pressure (PEEP) was applied for all with pressure between 4 and 10 cmH2O. Patients with multiple rib fracture accepted external rib fixation. Three received closed thoracic drainage because of pneumothorax or hemopneumothorax. Eight patients received surgeries, including 5 debridement suture and 3 open reduction internal fixation for extremity fracture. Six patients received hormone treatment, including 2 hormone bolus for spinal cord injury, 3 low-dose drip and 1 nebulization for pulmonary injuries. Fourteen patients got blood infusion due to loss of blood. Other treatment included external fixation of fracture or atlantoaxial subluxation, bronchofiberscope lavage, rehabilitation treatment, reduction of intracranial pressure by mannitol, and sedation.

No death occurred in our study. 47 (92.2%) patients were discharged without severe sequelae. The other 4 patients with sequelae included 3 patients of spinal cord injury and 1 patient of severe hypoxic-ischemic brain injury.

## Discussion

Traumatic asphyxia (TA), namely, perthes syndrome, thoracic compression syndrome, superior cava vena compression syndrome, has been rarely reported in literature, but its incidence is likely to be underestimated [6]. It was initially reported by Ollivier d’Angers in an entrapment accident in France, and it was further illustrated by Perthes with focus on neurological symptoms. Later, TA was found in car accident, object compression, entrapment or even without a large external force, such as crying, asthma, epilepsy. [1]

The recognized mechanism of TA was the counterforce between respiration and thorax, and increase of central venous system by thoracic compression and fear response [7], leading to superior vena cava (SVC) obstruction with incompetent valves to inhibit flowing back, arterial low perfusion and hypoxia, which could cause facial edema and cyanosis, neurological symptoms, petechiae of upper body and hemorrhage of conjunctiva, retina, ear, nose, oral mucosa, bronchi, etc. [1]

In our study, all patients showed petechiae on the upper body, especially on face. Twenty-five patients (49.0% CI 34.8–63.2%) out of 51 presented with facial edema. As for subconjunctival hemorrhage, 29 patients (56.9%, CI 42.8–70.9%) presented with subconjunctival hemorrhage. It is consistent with previous literature except that subconjunctival hemorrhage was of less percentage [8]. Compared to other existing literature were subconjunctival hemorrhage was seldomly absent [9], we got a much lower occurrence as 56.86%. This is possibly due to observation negligence or lacked medical recording by doctors who were not familiar with TA manifestations. Also, the incidence of facial petechiae was higher than previously reported, the reason for which might be that doctors are more possible to consider diagnosis of TA when encountering such characteristic appearance.

Hemorrhages of nose and ear were common in appearance and auxiliary examinations in this series. There were 7 mastoid hemorrhage and 10 hematocele of paranasal sinuses. Patients also showed nasal hemorrhage in 7, ear hemorrhage in 5, oral mucosa hemorrhage in 2 and hemoptysis in 1. Hemorrhage of mastoid and paranasal sinuses is possibly related to venous stasis and fear response through Eustachian tube and imbalanced pressure between middle ear and thorax with possible barotrauma [10]. As both brain and other region of head have veins belonging to SVC, it is reasonable to assume that hemorrhages out of brain could reduce the cerebral impairment as a protective reaction [10].

There are differing neurological symptoms as loss of consciousness, confusion, epileptic seizure [11]. In our study, 22 children underwent loss of consciousness within minutes or days, 3 children got confusion within days, and 25 remained conscious after injury. Six patients went through epileptic seizure, 5 vomited, and 11 got incontinence after injury, which may be correlated to both TA itself by circulatory abnormality and companion injuries as head bump. These symptoms revolved within days without surgical intervention except debridement suture. Only a patient with severe hypoxic-ischemic brain injury remained intellectual disability. Both respiratory inhibition and hemodynamic disturbances contribute to cerebral impairment in TA [11, 12]. However, their respective role is yet to be determined with not striking pathologic changes except congested vessel and petechial perivascular hemorrhage [13].

We want to highlight the evidence in viscera impairment of TA. There were autopsy evidence of subepicardial and subpleural petechiae [10]. Children got a less rigid thorax with intolerance to compression. Thus, the inner organs could suffer more from the compression with danger in cardiac and pulmonary impairment [14]. The potential mechanism of myocardial injury is the compression itself, the anoxia, and the cardiac capillary rupture due to venous hypertension as cardiac petechiae was found in TA victims [10]. We found a high percentage as 74.3%, indicating necessity in evaluation and intervention of cardia for TA patients. The mechanism of fear response, including deep inspiration, closure of the glottis, splinting of the thoracic and abdominal musculature, and chest compression, might provide protection to heart with elevated intra-thoracic pressure [15]. In addition, we found a percentage of hepatic insufficiency as 53.1% while the role of inferior vena cava (IVC) change in TA has been less focused in literature, even although there are evidence of increased pressure in IVC and visceral changes as “nutmeg” liver or liver petechiae during autopsy. [10, 16].

Also, the evaluation of retina through fundus is necessary. We found an incidence of 20.0% (CI 4.3–48.1%) from 15 patients in retinal hemorrhage, edema and exudates. Based on the result of 1 patient (Fig. 3), the retina was hemorrhagic with edema and exudates around the optic disc in the left eye. Moreover, the optic disc was pushed temporally, thus with optic nerve compression. The low-dosage dexamethasone was used as treatment for 3 days. However, in the 1-month and 1.5-month follow up, the retina still turned atrophy and optic disc was drafted nasally. It is reasonable to assume that the impairment is irreversible and could lead to lifelong amblyopia. Based on that, this evaluation is especially important for children as they are not capable to express feelings clearly and optic impairment could make its influence throughout their life [17].

As for other companion injuries, they are the main factors determining the prognosis and pulmonary contusion was the most frequent concomitant injury both in our study and other literature [18]. Pulmonary parenchymal injury results from several mechanisms, including direct compression, counter-coup compression, shearing forces and laceration by fractured ribs [19]. Interestingly, flail chest has not been found in our study. Accordingly, apart from suffocation, associated injuries are more causal in death instead of TA itself [20].

In addition, based on our study, there are 3 spinal cord injuries. Spinal cord injury deserves more attention as a severe injury correlating intimately with prognosis. Spinal cord injury was seldom reported with assumed injury mechanism as spinal anoxia and unstable blood flow [21]. However, we considered spinal injury as concomitant injury because as shown in Fig. 2, spinal cord injury often accompanies with spine fracture and spinal MRI usually showed spinal edema and abnormal signal from a specific segment, especially from the thorax.

No death occurred in our study, which is consistent with previous literature that TA got a rather good prognosis. This might be due to their thoracic wall elasticity [10]. Death of traumatic asphyxia is mostly caused by large compression force, compression overtime, and associated injuries [10]. The prognosis of TA depends on the severity of prognostic-related injuries.

### Limitations and strengths

As a retrospective study, the medical record might be imperfect because facing the rarely encountered TA by doctors from different departments, some symptoms might be neglected or misjudged. We failed to collect enough data about the forces causing traumatic asphyxia. There lacked accurate description of accident details as mechanism, time and patient’s state after injury. The examinations of myocardial markers, hepatic function, eye fundus and urine routine were not completely run for every patient. CT scan was not completed immediately after injury or admission, which not only interferes our analysis with possibly lower positive rates in statistics but also probably inhibit proper and timely treatment for concomitant injury.

Meanwhile, as a large and highly ranked medical center for children, we have collected 51 pediatric cases in TA, which is, from the acknowledgement of authors, of the largest sample ever. We also reported the rare OCT, fundus and thickness map results with comparison between admission and follow-up, thus drawing attention to the importance of retinal evaluation of TA.

## Conclusion

We reported 51 pediatric cases of TA which were mostly caused by traffic accident, object compression and stampede. Petechiae in facial, cervical and thoracic region, facial edema and cyanosis, subconjunctival hemorrhage and neurological symptoms were commonly found while the injury of heart, lung, liver, spinal cord and retina should not be neglected. Comprehensive physical and auxiliary examination should be performed after considering TA. No specific treatment other than supportive one was required for TA itself. The prognosis is rather good with timely intervention for companion injuries if central nervous system was not severely damaged.

## Data Availability

Statistical data are available from all authors but not available in public.

## Abbreviations

TA: Traumatic asphyxia
OCT: Optical coherent tomography
LOS: Length of stay
GCS: Glasgow coma score
PICU: Pediatric Intensive Care Unit
CI: Confidence interval
CK-MB: Creatine kinase isoenzyme-MB
cTn: Cardiac troponin
PEEP: Positive end expiratory pressure
SVC: Superior vena cava
IVC: Inferior vena cava
